# A Subset of CCL25-Induced Gut-Homing T Cells Affects Intestinal Immunity to Infection and Cancer

**DOI:** 10.3389/fimmu.2019.00271

**Published:** 2019-02-25

**Authors:** Hongmei Fu, Maryam Jangani, Aleesha Parmar, Guosu Wang, David Coe, Sarah Spear, Inga Sandrock, Melania Capasso, Mark Coles, Georgina Cornish, Helena Helmby, Federica M. Marelli-Berg

**Affiliations:** ^1^William Harvey Research Institute, Barts and The London School of Medicine and Dentistry, Queen Mary University of London, London, United Kingdom; ^2^Bart's Cancer Institute, Barts and The London School of Medicine and Dentistry, Queen Mary University of London, London, United Kingdom; ^3^Institute of Immunology, Hannover Medical School, Hannover, Germany; ^4^Kennedy Institute of Rheumatology, University of Oxford, Oxford, United Kingdom; ^5^Department for Immunology and Infection, London School of Hygiene and Tropical Medicine, London, United Kingdom

**Keywords:** chemokine, memory T cell differentiation, tissue resident cells, T helper (TH) 1 immunity, tissue microenvironment

## Abstract

Protective immunity relies upon differentiation of T cells into the appropriate subtype required to clear infections and efficient effector T cell localization to antigen-rich tissue. Recent studies have highlighted the role played by subpopulations of tissue-resident memory (T_RM_) T lymphocytes in the protection from invading pathogens. The intestinal mucosa and associated lymphoid tissue are densely populated by a variety of resident lymphocyte populations, including αβ and γδ CD8^+^ intraepithelial T lymphocytes (IELs) and CD4^+^ T cells. While the development of intestinal γδ CD8^+^ IELs has been extensively investigated, the origin and function of intestinal CD4^+^ T cells have not been clarified. We report that CCR9 signals delivered during naïve T cell priming promote the differentiation of a population of $\alpha_{4}\beta_{7}^{+}$ IFN-γ-producing memory CD4^+^ T cells, which displays a T_RM_ molecular signature, preferentially localizes to the gastrointestinal (GI) tract and associated lymphoid tissue and cannot be mobilized by remote antigenic challenge. We further show that this population shapes the immune microenvironment of GI tissue, thus affecting effector immunity in infection and cancer.

## Introduction

Immune surveillance of the gastrointestinal (GI) tract is conventionally thought to depend on the generation of a sizable cohort of recirculating $\alpha_{4}\beta_{7}^{\text{high}}$ memory T cells, which become able to access the gut parenchyma and gut–associated lymphoid tissue (GALT).

The gut wall is densely populated by a variety of resident immune cells required for effective immune responses against pathogens, while allowing coexistence with commensals and preventing autoimmunity. For example, intraepithelial αβ and γδ CD8^+^ T lymphocytes (IELs) reside within the intestinal epithelial layer provide a first line of defense at this extensive barrier (1). A substantial cohort of memory CD4^+^ T cells is also present in the intestinal wall, particularly in the *lamina propria* (LP) (2). Most of these cells display a Th1 phenotype in mice and humans (3–5). LP CD4^+^ T cells also bear a distinctive homing phenotype, including co-expression of α_4_β_7_ and CCR9 (6). While the ontogenesis of TCR-αβ/γδ CD8αα intraepithelial T lymphocytes (IELs) has been extensively investigated (7), the origin and function of this CD4^+^ T cell subset remain unclear (8).

Tissue-derived factors play a key role in the differentiation of T cells that populate non-lymphoid tissue, including tissue-resident memory (T_RM_) T cells, which arise during priming, reside long-term in tissues and play a key role in local protection from re-infections (9). For example, the CXC-chemokine receptor 3 (CXCR3) is required for the localization of effector T cells to the epidermis and for subsequent T_RM_ cell differentiation (10). Similarly, CXCR3 is instrumental for the localization of effector T cells to the lung epithelium (11, 12). In the intestine, genetic deletion of CCL25 or its receptor CCR9 results in depletion of IELs (13, 14), which was attributed to impaired ability of these T cells to localize to the gut wall. CCL25 expression is enhanced in inflamed intestine (15), suggesting that its availability in GALT increases during immune activation and the generation of immunological memory.

Based on these observations, we have investigated the contribution of the CCR9-CCL25 axis to the generation and function of CD4^+^ T cell-mediated immunological memory in the intestine and associated lymphoid tissue. We show that CCR9 signals during priming promote the development of a Th1 population with features of T_RM_ cell which regulates the local immune environment and protective responses against GI infections and tumors.

## Materials and Methods

### Mice

Mice were used at the age of 7–11 weeks. C57BL/6 mice were purchased from Charles River (UK). Female Marilyn mice, bearing a transgenic TCR specific for the male minor transplantation antigen HY peptide epitope *Dby* (NAGFNSNRANSSRSS) and restricted by H2-A^b^ molecules, have been previously described (16). In this study, Marilyn-Rag2^−/−^ mice obtained by backcrossing for nine generations were used. *ccl25*^−/−^ and *ccr9*^−/−^ mice have been previously described (13, 14). OT-II mice, bearing a transgenic TCR specific for the ovalbumin peptide epitope OVA_323−339_ (ISQAVHAAHAEINEAGR) and restricted by H2-A^b^ molecules, have been previously described (4). All the *in vivo* experiments were conducted under the Home Office regulation and approved by the local Ethics Committee.

### Reagents

The cell linker PKH26 was purchased from Sigma-Aldrich and used at 2 μM. CFSE was purchased from Invitrogen and used at 4 μM. Dylight 488 Amine-Reactive Dye and Kits were purchased from Thermo Scientific. In proliferation assays measuring CFSE dilution by flow cytometry, the average number of cell divisions that a cell in the original population has undergone (Division Index) was measured using Flowjo 7.6 (TreeStar Inc).

The chemokine CCL25 was purchased from PeproTech EC Ltd. The Dby peptide was purchased from Cambridge Bioscience. Pertussis Toxin was purchased from Sigma. 3,7-dimethyl-2,6-octadienal (Citral) was purchased from Sigma and used in the co-cultures at a working concentration of 0.1 μM.

### Antibodies

Naïve T cells were purified by immunomagnetic negative selection using EasySep™. Mouse Naïve T cells Isolation Kits (Stemcell Technologies) according to manufacturer's instructions.

The affinity-purified polyclonal goat anti-mouse CCR9 Ab was purchased from Novus Biological (NB100-708). The immunogen for this antibody is the peptide IPGMFDDFSYDSTASTDDYMNLNFSSFF, corresponding to amino acids 10–37 of Mouse CCR9. Its biological activity has not been described.

For immunohistochemistry, the following antibodies were used: Armenian hamster anti-mouse **C**D11c (1:50, clone N418, BioLegend), polyclonal Rat anti-mouse CD31 Antibody (clone MEC 13.3, Cat No: 102502, BioLegend), rat anti-mouse CCL25 Ab (clone 89827, R&D), Rat anti-mouse MadCAM-1 (clone: MECA-367, Cat No: 16-5997-85, ThermoFisher). Alexa Fluor 555-conjugated Goat Anti-Rat IgG (H+L) (1:100), and Alexa Fluor 488-conjugated Goat Anti-Hamster IgG (H+L) (1:100) were purchased from Invitrogen/Life Technologies.

All flow cytometry antibodies were used at 1:200 dilution unless otherwise specified. APC-conjugated anti-mouse α_4_β_7_ (clone DATK32), PE-conjugated anti-mouse CCR9 (clone CW-1.2), PerCP-eFluor® 710-conjugated anti-mouse IL-4 (clone 11B11), eFluor® 450-conjugated anti-mouse IL-17A (clone eBio17B7), PE-conjugated anti-mouse IL-17A (clone eBio17B7), PE-conjugated anti-mouse anti-mouse T-bet (Clone eBio4B10 (4B10, 4-B10), PE-conjugated anti-mouse Gata-3 (Clone TWAJ), FITC-conjugated anti-mouse CD4 (clone GK1.5), eFluor® 450-conjugated anti-mouse CD4 (clone RM4-5), APC-eFluor® 780-conjugated anti-mouse CD8 (clone 53-6.7), APC-conjugated anti-mouse FoxP3 (clone FJK-16s), PerCP-eFluor® 710-conjugated anti-mouse Vβ6 TCR (clone RR4-7), eFluor® 450-conjugated anti-mouse Ly-6C (clone HK1.4), PerCP-Cyanine5.5-conjugated anti-mouse CD19 [clone eBio1D3 (1D3)], PE-conjugated anti-mouse F4/80 (clone BM8), FITC-conjugated anti-mouse CD11c (clone N418) were purchased from eBioscience. PE/Cy7-conjugated anti-mouse CD3 (clone 145-2C11), Brilliant Violet 785™-conjugated anti-mouse CD45 (clone 30-F11), Brilliant Violet 605™-conjugated anti-mouse CD11b (clone M1/70), Alexa Fluor® 700-conjugated anti-mouse Ly-6G (clone 1A8) were purchased from BioLegend. The LIVE/DEAD™ Fixable Aqua Dead Cell Stain (Cat L34957) were purchased from ThermoFisher. Rabbit anti-mouse Phospho-Akt (Ser473) (clone 193H12) was purchased from Cell Signaling. Mouse neutralizing CCL25/TECK MAb (Clone 89818) was purchased from R&D. Purified polyclonal goat anti-mouse CCR9 Ab (NB100-708) was purchased from Novus.

For accuracy, in flow cytometric analysis of cells experiments, gating settings based on staining with isotype-matched control antibodies were made on a 1:1 mixture of cells from experimental and control animals.

### Generation of Bone Marrow-Derived DCs

Bone marrow (BM)-derived DCs were obtained from WT C57BL/6 (H2-b) mice. Femurs from 7-to-10-week-old female mice were removed and BM cells were flushed out with PBS using a 27-gauge needle (Fischer Scientific, Loughborough, UK). Red blood cells were lysed from the cell suspension with lysis buffer (Sigma). BM cells (5 × 10^6^) were seeded per well in a six well plate in RPMI 1640 medium supplemented with 10% FCS, 2 mM glutamine, 50 IU/mL penicillin, 50 μg/mL streptomycin, 50 μM 2-ME, and 2% murine granulocyte-macrophage colony stimulating factor (GM-CSF) obtained from the supernatant of the GMCSF hybridoma (gift from Dr. Jian-Guo Chai, King's College, London, UK). Cells were cultured at 37°C in the presence of 5% CO2. On days 3 and 5, fresh culture medium was added to the plates. DCs were matured overnight with 100 ng/ml lipopolysaccharide (LPS) and used between 7 and 10 days post-isolation.

### Measurement of RALDH Activity in DCs

Cell aldehyde dehydrogenase (ALDH) activity was determined by using the ALDEFLUOR staining kit (Stemcell Technologies, Cambridge, UK) according to the manufacturer's instructions. In brief, DCs treated with or without 0.1 μM citral were resuspended at 10^6^ cells/ml in ALDEFLUOR assay buffer containing activated ALDEFLUOR substrate and incubated at 37°C for 30 min. ALDH inhibitor diethylaminobenzaldehyde (DEAB) was used as negative control. ALDEFLUOR-reactive cells were detected by flow cytometry in the FITC channel.

### Intracellular Cytokine Staining

For stimulating cytokine production, T cells (2 × 10^6^/mL) were re-stimulated with irradiated unpulsed or Dby (Ab HY epitope) peptide-pulsed B6 female T cell–depleted splenocytes (4 × 10^6^/mL) in 24-well plates for 6 h in the presence of monensin (GolgiStop; BD Biosciences Pharmingen). Cultured cells were stained with antibodies for surface molecules. After washing, cells were fixed, permeabilized with Cytofix/Cytoperm solution (BD Biosciences Pharmingen), washed and resuspended in 1X Perm/Wash solution (BD Biosciences Pharmingen) containing antibodies for intracellular cytokines or isotype-matched control antibodies. After a final wash, the cells were resuspended in staining buffer for flow cytometric analysis.

### Wide Field Deconvolution Fluorescence Microscopy and Flow Cytometry

Tissue samples were either processed for flow cytometric analysis (lymph nodes and spleen) or embedded in Optimal Cutting Temperature compound (OCT, Agar Scientific Ltd, UK), snap-frozen and stored until analysis.

Frozen tissue sections were laid onto Polylisine Microscope slides (VWR International Lutterworth, Leicestershire, UK), left to dry overnight, and then mounted in Vectorshield mounting medium for fluorescence with DAPI (Vector Laboratories, Peterborough), to visualize the nuclei. Slides were visualized with a Coolview 12-cooled CCD camera (Photonic Science, Newbury, UK) mounted over a Zeiss Axiovert S100 microscope equipped with Metamorph software (Zeiss, Welwyn Garden City, UK). A × 10 and NA 0.6 objectives and standard epi-illuminating fluorescein and rhodamine fluorescence filter cubes were used and 12-bit image data sets were generated. Tissue infiltration was quantified by randomly selecting ten 10x-magnified fields and assessing the number of fluorescent cells in each field, as previously described (17). Quantification of T cell infiltrates observed by wide field fluorescence microscopy was performed using a purpose-designed software to run in the LabView (V7.1, National Instruments) environment (17), which allows identifying single fluorescent cells within the tissue. To minimize the effect of arbitrary choice of field, tissue infiltration was quantified by randomly selecting ten 10x-magnified fields from 3 to 6 tissue samples of each tissue. The number of infiltrating cells obtained was then averaged and assessed statistically. Infiltration is expressed as the mean of fluorescent cells per ten 10x field in a given experimental condition.

Flow cytometric analysis was performed using a LSR FORTESSA (Becton Dickinson, Mountain View, CA) and FlowJo version 7.6.5 software (Tree Star Inc, Ashland, OR, USA).

### Measurement of AKT/PKB Phosphorylation

Naïve T cells and DCs were stimulated with 300 ng/ml CCL25 and harvested at the indicated time points, fixed with 2% PFA for 15 min at 37°C, washed twice with PBS, permeabilized with 90% ice-cold methanol for 10 min at −20°C then washed twice with PBS. Intracellular staining was carried out after initially blocking permeabilized cells in RT FACS buffer (0.5% BSA/PBS + Na3VO4) for 30 min and incubation with a dilution of 1:300 of Phospho-Akt (Ser473) rabbit anti-mouse antibody (Cell Signaling Technology) for 30 min at room temperature. Cells were washed and stained with secondary APC-F(ab)_2_ fragment donkey anti-rabbit IgG (H+L) (Jackson ImmunoResearch, Suffolk, UK) at 1:300 for 30 min at room temperature. Cells were then analyzed using flow cytometry.

## DCs Staining With Labeled CCL25

DCs were incubated with Dylight 488-labeled (300 ng/ml, Thermo Scientific, UK) CCL25 or albumin (control) for 40 min, followed by washing in PBS. One hundred and fifty microliters of Dylight 488-CCL25 treated DCs were spread onto a slide coated with poly-l-lysine (Sigma). Slides were incubated for 25 min at 37°C, followed staining with 1:50 hamster anti-mouse CD11c antibody (clone N418, BioLegend). Samples were fixed in 4% paraformaldehyde for 10 min at 37°C. Following three washes in PBS, the sections were incubated with the secondary antibody Alexa Fluor® 555 goat anti-hamster IgG (Life Technologies) for 30 min at room temperature followed by three washes. Sections were mounted on microscopy slides with 4′,6-diamidino-2-phenylindole (DAPI) mounting medium (Vectashield). Alternatively, cells were analyzed by flow cytometry.

### Helminth Infestation Model

Six to eight week old C57BL/6 mice were obtained from Charles River UK Ltd (Margate Kent, UK). All experiments were performed at least twice and the sizes of the experimental groups were 3–5 mice per group per time point. Experimental animals were infected with 150 embryonated *Nippostrongylus brasiliensis* eggs on day 0 by oral gavage. Maintenance and recovery of the parasites were conducted as described previously (18). To assess cytokine production, mesenteric lymph nodes (MLN) and spleens were removed from uninfected and infected animals and single cell preparations were resuspended in RPMI 1,640 supplemented with 10% heat-inactivated FCS, 2 μM L-glutamine, 100 U/ml penicillin, 100 mg/ml streptomycin and 0.05 μM 2-mercaptoethanol (all from Life technologies, Paisley, UK). Cells were cultured at 37°C and 5% CO2 in flat-bottomed 96-well plates (Nunc, Roskilde, Denmark) at a final concentration of 5 × 10^6^/ml in a final volume of 0.2 ml/well. Cells were stimulated with *N. brasiliensis* ES antigen (25 μg/ml) or plate-bound anti-CD3 antibody (mAb145-2C11, 10 μg/ml, ATCC). Cell free supernatants were harvested after 48 h and stored at −80°C. Cytokine analyses were carried out using commercially available sandwich ELISAs for IL-4, IFN-γ (Mabtech AB, Nacka, Sweden), IL-13 and IL-10 (R&D systems, Abingdon, UK).

### Pancreatic Ductal Adenocarcinoma Model

The KPC pancreatic ductal adenocarcinoma (PDAC) cell line was derived from a KPC mouse generated from crossing the LSL-Kras-G12D, LSL-p53-R172H, and Pdx-1-Cre mice (NCI, Frederick, MD). Mice were immunized i.p. with 5 × 10^5^ KPC cells, which had previously undergone 5 rounds of freeze-thawing in liquid nitrogen, mixed with 5 × 10^6^ autologous splenocytes in a volume of 100 μl PBS, with and without 0.06 mg/kg CCL25. For orthotopic implantation of KPC cells into the pancreas of C57BL/6 mice, while the mice were anesthetized, a small incision was made in the skin and peritoneal lining and the pancreas externalized. Using a Hamilton® syringe, 700 series, removable needle volume 25 μl, needle size 22 s gauge (bevel tip) (#10100332, Fisher Scientific), ~5 × 10^5^ KPC cells in a volume of 5 μl PBS were injected into the body of the pancreas. Mice were checked daily and the experiment was terminated 28 days post orthotopic surgery. Tumors were weighed after excision and processed for imaging or flow cytometry.

### Processing of Tumor Tissue

Pancreatic tumors were minced using a sterile scalpel blade and single cell suspensions were generated using enzymatic digestion (1 mg/ml Collagenase D, and 10 μg/ml DNAse 1). Tumor debris was removed using 70 micron filters (BD Falcon) before staining for flow cytometry or RNA extraction.

### Quantitative Real-Time Polymerase-Chain Reaction (qRT-PCR)

Tissues were harvested and stored at −80°C until processing. Tissues were sliced and homogenized with stainless steel bead using TissueLyser. RNA was purified using Qiagen RNAeasy Kit according to the manufacturer's instructions. Reverse transcription was performed according to the manufacturer's instruction (Applied Biosystems). Gene expression analysis was done using SYBR Green Supermix (Biroad) in CFX connect light cycler (Biorad), according to the manufacturer's instructions. Primer sequences are below:

**Table:** 

**Gene**	**Forward sequence**	**Reverse sequence**
GAPDH	GGCAAATTCAACGGCACAGT	AGATGGTGATGGGCTTCCC
HPRT	GTAATGATCCAGTCAACGGGGGAC	CCAGCAAGCTTGCAACCTTAACCA
IFN-γ	TCAAGTGGCATAGATGTGGAAGAA	TGGCTCTGCAGGATTTTCATG
IL-5	GCCAGCGCTGAAGACTTC	CTTGTCAACAGAGCTCGGTG
IL-17	TTTTCAGCAAGGAATGTGGA	TTCATTGTGGAGGGC AGAC
TGF-β	CCATCCATGACATGAACCGG	TGGTATCCAGGGCTCTCC
CCL8	CAGTCACCTGCTGCTTTCAT	ACAGCTTCCATGGGGCAC
CCL10	CCCTCTCCTTCCTCATTCTTACA	AGTCTTGAAAGCCCATGTGAAA
IL1-β	TGAGCTTTGTACAAGGAGAACC	GGTGTGCCGTCTTTCATTACA

### Statistical Analysis

Results are expressed as mean standard error of the mean (SEM) or mean SD. The Student's *t*-Test and ANOVA test were used. All reported *p*-values are two-sided. A *p* value of < 0.05 was regarded as significant.

## Results

### Activated CCR9^+^ CD4^+^ T Cells Acquire α_4_β_7_ Integrin Expression *in vitro* in the Presence of DC-Bound CCL25

Following activation in the draining lymph nodes, T cells localize to non-lymphoid tissue by expressing appropriate homing receptors. We therefore began our investigation by evaluating the effect of exposure to CCL25 during activation on the expression of the gut homing receptor α_4_β_7_ during the generation of T cell memory by a series of *in vitro* experiments. In our hands, the CCL25 receptor CCR9 is expressed in ~70% of CD8^+^ and 4–15% of CD4^+^ naïve T cells (CD44^low^CD62L^bright^, Supplementary Figures 1A,B), in line with previous reports (19). A series of preliminary experiments using antibody-activated naïve T cells showed that the presence of CCL25 optimally upregulated α_4_β_7_ expression by WT, but not CCR9-deficient naïve T cells. This effect was observed only when CCL25 was delivered to the cultures bound to autologous DCs rather than in soluble form and when T cells were allowed to directly interact with the DCs in the cultures (Supplementary Figures 1C,D). As DC differentiation in GM-CSF enhances retinaldehyde dehydrogenase (RALDH) activity and production of retinoic acid (20), which in turn induces gut-homing receptors (21), to detect CCL25-specific effects DCs were pre-treated with the RALDH inhibitor 3,7-dimethyl-2,6-octadienal(Citral) (Supplementary Figures 1E,F).

**Figure 1:**
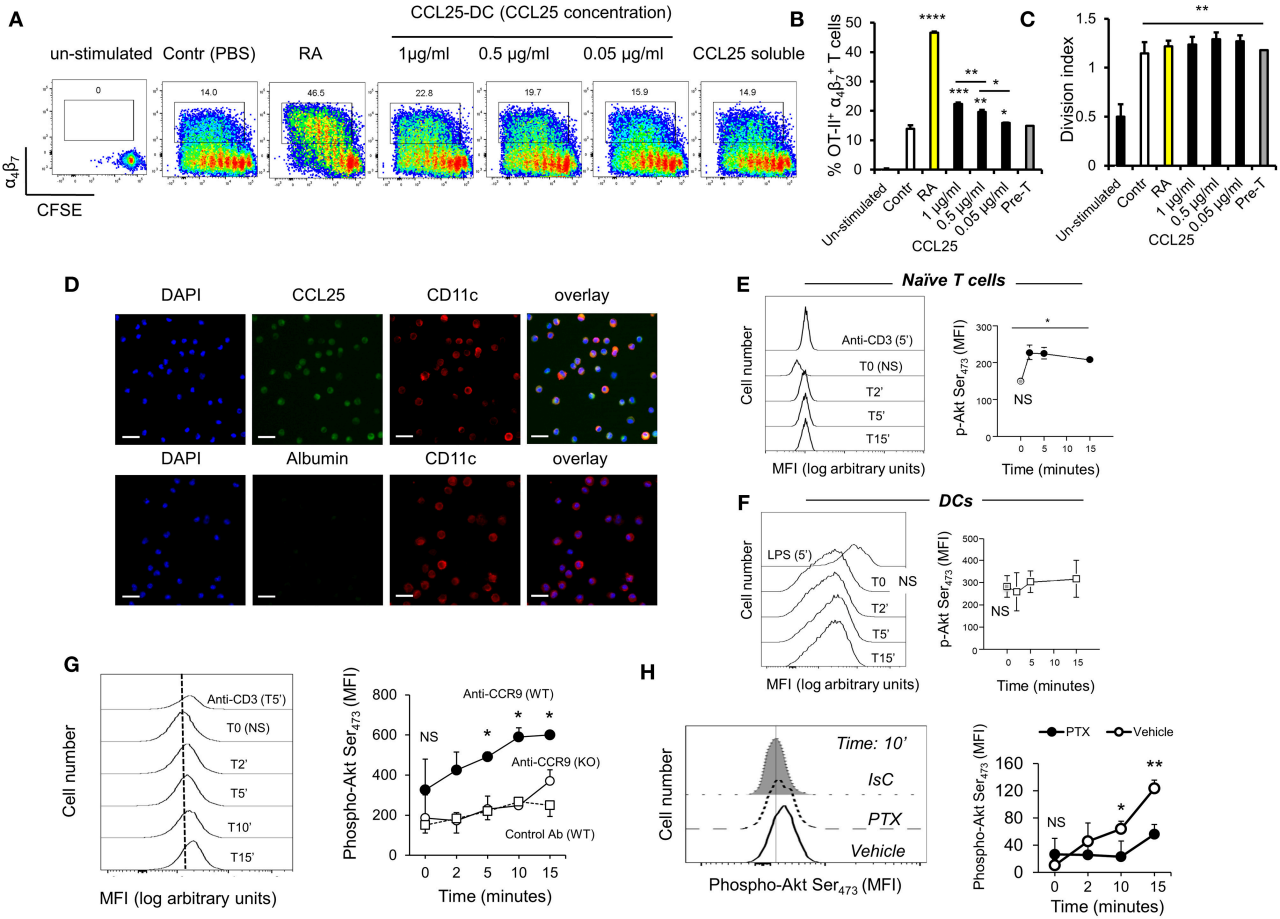
CCR9 signals promote the acquisition of gut-homing receptors during *in vitro* T cell activation. **(A–C)** OT-II T cells were labeled with CFSE (4 μM) and stimulated with OVA_323−339_-pulsed mature C57/Bl6 bone-marrow (BM)-derived DCs pre-treated with increasing concentrations of CCL25 (pre-DC) or PBS alone. As a control, T cells were activated in the presence of retinoic acid (RA) or pre-treated with CCL25 and subsequently exposed to untreated antigen-presenting DCs (pre-T). After 3 days, expression of the integrin α_4_β_7_ by CFSE^low^ T cells was assessed by flow cytometry. The bar graph shows the mean data from three experiment of identical design each performed in triplicate (±SEM). In panel **(C)**, the stimulation index calculated for the different cultures is shown (*N* = 3). Asterisks over each column bar represent comparison with control. Asterisks over lines represent comparisons between the columns included between the lines. **(D)** Representative confocal microscopy image of non-permeabilized DCs incubated with green Dylight 488-labeled CCL25 (300 ng/ml) or albumin (control) and counter-stained with Hamster-anti-mouse CD11c. Scale bar = 40 μm. **(E,F)** Naïve T cells **(E)** or DCs **(F)** were exposed to CCL25 (300 ng/ml) for up to 15 min and phosphorylation of Akt at serine 473 was assessed by antibody staining and flow cytometry. Representative histograms of the experimental conditions indicated beside each profile are shown in the left-hand panels. pAktSer_473_ expression by non-stimulated T cells or DCs (T0, NS), and by T cells and DCs stimulated for 5 min with anti-CD3 (1μg/ml) or LPS (100 ng/ml), respectively, are shown. The right-hand panels show the mean fluorescence intensity (MFI) indicative of Akt phosphorylation obtained in 3 experiment of identical design each performed in triplicate (±SEM, ^*^p < 0.05 vs. NS). **(G,H)** Purified naïve T cells were exposed to the NB100-708 anti-CCR9 antibody (5 μg/ml) cross-linked with 2.5 μg/mL Donkey–anti-goat IgG for up to 15 min and phosphorylation of Akt at serine 473 (pAkt_Ser473_) was assessed by flow cytometry. **(G)** Representative histograms of T cells stained with the pAkt-specific antibody are shown in the left-hand panel. pAkt_Ser473_ expression after 5-min stimulation with anti-CD3 is shown. The mean MFI values obtained at the indicated time points obtained in three experiments of identical design each performed in triplicates is shown on the right-hand panel (±SD, ^*^p < 0.05, ^**^p < 0.01 vs. time 0, NS), which includes pAkt_Ser473_ expression by CCR9-competent T cells in the presence an Isotype-matched antibody (IsC) (WT) and by *ccr9*^−/−^ T cells (KO) incubated with the anti-CCR9 Ab. **(H)** In some T cells CCR9 was antibody-activated in the presence of pertussis toxin (PTX, 0.1 μg/ml). A histogram displaying pAkt_Ser473_ measured at 10 min after stimulation is shown on the left hand side. The mean MFI values obtained in three experiments of identical design each performed in triplicates are shown in the right-hand panel (±SD). ^*^p < 0.05, ^**^p < 0.01, ^***^p < 0.001, ^****^p < 0.001.

To confirm these observations, CFSE-labeled ovalbumin (OVA)-specific TCR-transgenic OT-II T cells were stimulated by antigen-pulsed DCs pre-exposed to increasing concentration of CCL25, which led a proportional increase of α_4_β_7_-expressing T cells in the divided population (Figures 1A,B). The presence of CCL25 however did not affect the rate of T cell division (Figure 1C).

With the exception of a tolerogenic plasmacytoid subset, DCs do not express CCR9 (22). However, confocal imaging of CCR9-depleted bone marrow-derived DCs exposed to labeled CCL25 (or Albumin as a control) revealed that the chemokine was bound to a large proportion of DCs (Figure 1D), indicating that DC immobilize CCL25 independently of CCR9 expression. Further experiments confirmed that binding of CCL25 to DCs does not elicit Akt activation, while small but consistent Akt phosphorylation (comparable to that induced by CD3 antibody-triggering) occurred in naïve T cells exposed to the chemokine (Figures 1E,F). The ability of CCR9 to elicit signals in naïve T cells was confirmed by the induction of Akt phosphorylation by a stimulatory anti-CCR9 antibody (Figure 1G), which was prevented by exposure of the T cells to pertussis toxin (PTX, Figure 1H).

### CCL25 Promotes the Differentiation of a Population of $\alpha_{4}\beta_{7}^{\text{bright}}$ CD4^+^ T Cells *in vivo*

We next sought to establish the contribution of CCL25 to the differentiation of CD4^+^ $\alpha_{4}\beta_{7}^{\text{bright}}$ T cells in physiologic conditions during oral immunization.

First, we analyzed the localization of this chemokine in healthy murine gut parenchyma and GALT. Endogenous CCL25 was present in the gut *lamina propria* (LP) and Peyer's patches (PP) where it co-localized with CD11c^+^ cells, but not in mesenteric and other LNs (Supplementary Figure 2A) in steady state conditions. CCL25 was found on the vessels in the LP, but not in GALT (Supplementary Figure 2B). Following induction of inflammation, CCL25 was retrieved on CD11c^+^ cells also in the mesenteric LNs (Supplementary Figure 2C), but not on high endothelial venules (HEV, Supplementary Figure 2D), suggesting that gut-produced CCL25 is transported to the draining LNs via CD11c^+^ cells (likely migratory tissue-derived DCs)—as unbound CCL25 directly transported to the LN would diffuse and bind to HEV.

**Figure 2:**
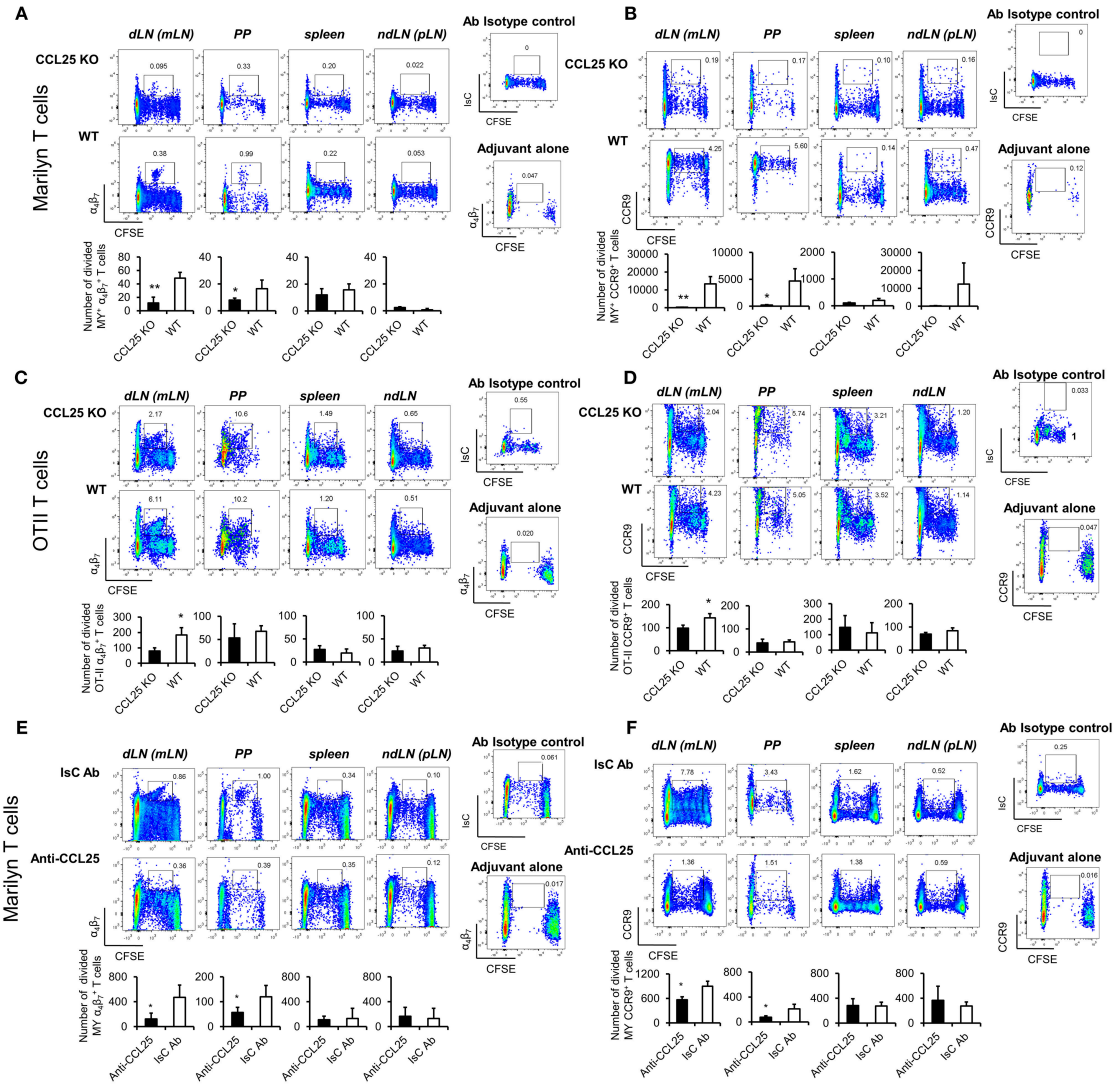
CCL25 promotes differentiation of an antigen-specific, $\alpha_{4}\beta_{7}^{\text{high}}$ CD4^+^ T cell population. TCR-transgenic, MY **(A,B)** or OT-II **(C,D)** naïve T cells (10^7^/mouse) were labeled with CFSE (4 μM) and injected intravenously into WT and *ccl25*^−/−^ syngeneic female recipients. Twenty-four hours later, recipient mice received cognate peptide (100 μg Dby or 0.5 μg OVA-DEC peptide) plus ODN adjuvant (50 μg/mouse) orally. As a control, some mice received adjuvant only (ODN alone). Five days after immunization, T cells were separately harvested from mesenteric LN (draining LN, dLN), Peyer's Patches (PP), inguinal and axillary (non-draining LNs, ndLN) and the spleen. T cells were identified by gating on the CD4^+^ Vβ6^+^ (MY) or Vβ2^+^ (OTII) lymphocyte populations. Expression of α_4_β_7_ **(A,C)** and CCR9 **(B,D)** by divided T cells was assessed by flow cytometry. In similar experiments depicted in panels **(E,F)**, CD4^+^ T cells from Marilyn mice (MY 10^7^/mouse) were labeled with CFSE (4 μM) and adoptively transferred into syngeneic female recipients, which received an intraperitoneal injection of CCL25-neutralizing antibody (Anti-CCL25, 10 mg/kg) or Isotype Control antibody (IsC Ab). Twenty-four hours later, recipient mice received cognate Dby peptide (100 μg) plus ODN adjuvant (50 μg/mouse) orally. Five days after immunization, T cells were separately harvested from mesenteric LN (draining LN, dLN), Peyer's Patches (PP), inguinal and axillary (non-draining LNs, ndLN) and the spleen. T cells were identified by gating on the CD4^+^ Vβ6^+^ (MY) lymphocyte populations. Expression of α_4_β_7_ **(E)** and CCR9 **(F)** by divided T cells was assessed by flow cytometry. In all panels, the mean number of divided $\alpha_{4}\beta_{7}^{+}$ and CCR9^+^ T cells from three independent experiments of identical design is shown below a set of representative dot plots (±SD). Staining with an isotype-matched control antibody and proliferation and differentiation in draining lymph nodes of mice injected with adjuvant alone are shown on the right-hand side of each set of dot plots. To avoid the reciprocal effect of the presence of other responder T cell populations, results are presented as absolute numbers. ^*^p < 0.05, ^**^p < 0.01.

Next, purified naïve TCR-transgenic Marilyn (MY, male antigen HY-specific H2-A^b^-restricted, Figures 2A,B) or OT-II (Ovalbumin-specific, H2-A^b^-restricted, Figures 2C,D) T cells were labeled with CFSE, and adoptively transferred into female *ccl25*^−/−^ and WT recipients (H2-b haplotype), which were subsequently immunized orally with cognate peptide in 50 μg of CpG ODN adjuvant. Five days later T cells were isolated from draining mesenteric lymph nodes (MLN, dLN), Peyer's patches (PP), spleen, and non-draining LNs (ndLN, inguinal and axillary). Expression of α_4_β_7_ and CCR9 by T cells that underwent division was monitored by flow cytometry. As shown in Figure 2, discrete populations of $\alpha_{4}\beta_{7}^{\text{high}}$ (Figures 2A,C) and CCR9^+^ (Figures 2B,D) T cells differentiated within dividing TCR-transgenic T cells in dLN following oral immunization. These populations were significantly reduced in *ccl25*^−/−^ recipients. Interestingly, while CCR9 upregulation occurred in most proliferating T cells, only a small fraction of α_4_β_7_-expressing T cells was consistently detected at the 4–5th round of division. Administration of adjuvant alone did not lead to cell division or upregulation α_4_β_7_. All α_4_β_7_-expressing T cells were also CCR9^+^ (Supplementary Figure 2E).

In parallel experiments, treatment with a neutralizing CCL25 antibody significantly reduced the development of $\alpha_{4}\beta_{7}^{+}$CCR9^+^ T cells in dividing MY T lymphocytes transferred into syngeneic C57BL/6 female (23) during priming (Figures 2E,F). The role of CCR9 signals in the development of this T cell population was further confirmed in similar experiments in which lack of CCR9 expression by adoptively transferred T cells resulted in a significantly reduced number of divided T cells expressing $\alpha_{4}\beta_{7}^{+}$ in the GALT in response to allo-immunization (Supplementary Figure 2F).

### CCR9 Signals Promote the Development of a Th1-Like Phenotype by Inducing Expression of the Transcription Factor T-Bet

**Figure 3:**
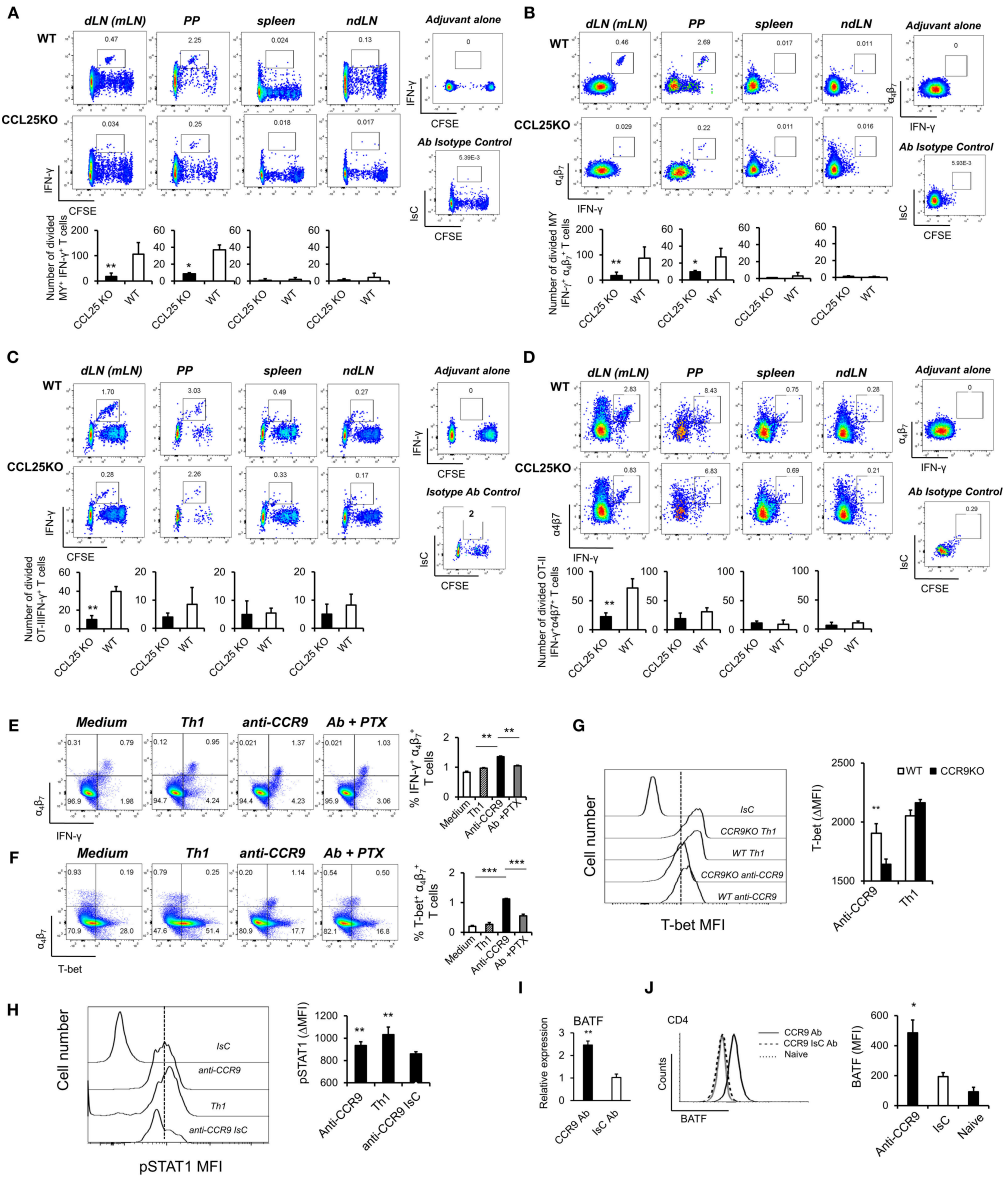
CCR9 signals induce $\alpha_{4}\beta_{7}^{+}$ Th1-like cells. MY **(A,B)** or OT-II **(C,D)** naïve T cells (10^7^/mouse) were labeled with CFSE (4 μM) and injected intravenously into WT and *ccl25*^−/−^ syngeneic female recipients. Recipient mice received cognate peptide (100 μg Dby or 0.5 μg OVA-DEC peptide) plus ODN adjuvant (50 μg) orally 24 h later. Five days later, T cells were separately harvested from mesenteric LN (draining LN, dLN), inguinal and axillary (non-draining LNs, ndLN) and the spleen. Production of IFN-γ by divided MY **(A)** and OT-II **(C)** T cells was assessed by intracellular staining and flow cytometry. T cells were identified by gating on the CD4^+^ Vβ6^+^ (MY) or Vα2^+^ (OTII) populations. Panels **(C)** and **(D)** show the production of IFN-γ by $\alpha_{4}\beta_{7}^{+}$ divided MY an OTII T cells, respectively. The mean number of T cells from three independent experiments of identical design is shown below each set of representative dot plots (±SD, *n* = 3, *N* = 2). **(E,F)** Naive T cells were activated either with plastic bound anti-CD3 in culture medium, or under Th1-skewing culture conditions or in the presence of an activating CCR9 antibody (7 μg/ml). In some experiments, T cells were activated in the presence of the anti-CCR9 antibody in the presence of pertussis toxin (PTX, 0.1 μg/ml). Representative dot-plots of IFN-$\gamma^{+}\alpha_{4}\beta_{7}^{+}$ and T-${\text{bet}}^{+}\alpha_{4}\beta_{7}^{+}$ T cells (gated on CD3^+^CD4^+^ populations) 5 days after activation and the mean number of T cells from three independent experiments of similar design is shown in panels **(E,F)**, respectively (±SD). Of note, Th1-skewing culture led to T-bet expression mainly in $\alpha_{4}\beta_{7}^{-}$ T cells **(F)**. **(G)** WT and *ccr9*^−/−^ naive T cells were activated either with plastic bound anti-CD3 under Th1-skewing culture conditions or in the presence of an activating CCR9 antibody (7 μg/ml). Five days later, expression of T-bet was assessed by flow cytometry. A representative histogram and the mean T-bet MFI from three independent experiments of similar design are shown (±SD). **(H)** Naive T cells were activated either with plastic bound anti-CD3 and antiCD28 under Th1-skewing culture conditions or in the presence of an activating CCR9 antibody (7 μg/ml) or isotype-matched antibody control. Three days later, expression of phosphorylated (p)STAT1 (Ser_727_) was assessed by flow cytometry. Representative histogram and the mean pSTAT1 MFI from three independent experiments of similar design are shown (±SD). **(I,J)** Naïve WT T cells were stimulated with plate-bound anti-CD3 and CD28 antibodies in the presence of either 7 μg/mL anti-CCR9 or isotype control antibody cross-linked with 2.5 μg/mL Donkey–anti-goat IgG overnight. T cells were harvested and expression of BAFT mRNA **(I)** protein **(J)** by CD4^+^ T cells was assessed by real-time PCR and intracellular staining, respectively. Staining with an isotype-matched control antibody and proliferation and differentiation in draining lymph nodes of mice injected with adjuvant alone are shown on the right-hand side of each set of dot plots. Bar graph show the mean values obtained in three experiments of identical design each performed in triplicates (±SD). ^*^p < 0.05, ^**^p < 0.01, ^***^p < 0.001, ^****^p < 0.0001.

The experiments described above also revealed that IFN-γ production by $\alpha_{4}\beta_{7}^{+}$ MY and OT-II primed T cells was impaired in CCL25-deficient mice immunized orally (Figures 3A–D). Differentiation of IL-4- or IL-17-producing T cells in the proliferating populations appeared unaffected (Supplementary Figures 3A,B).

To further explore this observation, cultures were set up to assess the effect of CCR9 signals on T-bet expression. A stimulatory anti-CCR9 antibody (Figures 1G,H) capable of mimicking the effects of CCL25 on CD4^+^ T cell differentiation *in vivo* (Supplementary Figures 3C,D) was used in these experiments, as induction of T cell differentiation by CCL25 requires the presence of DCs, which might provide additional T-bet-inducing signals. T cells were stimulated with plate-bound anti-CD3 in the presence of the CCR9-stimulating or isotype-matched control antibody. As a positive control for Th1 differentiation, T cells were activated in the presence of IL-12 and anti-IL-4 mAb. Antibody triggering of CCR9 during activation with anti-CD3 led to a significant increase of IFN-γ-producing $\alpha_{4}\beta_{7}^{+}$ T cells (Figure 3E). Importantly, enhanced production of IFN-γ was accompanied by expression of the Th1-associated transcription factor T-bet selectively in α_4_β_7_-expressing T cells (Figure 3F). This, in turn, was inhibited by treatment with pertussis toxin (PTX), confirming that CCR9 signals directly and selectively promote the differentiation of Th1 properties in $\alpha_{4}\beta_{7}^{+}$ T cells. Control experiments were conducted with CCR9-deficient T cells, which failed to upregulate T-bet in response to the CCR9-stimulating antibody (Figure 3G). Additionally, antibody-activation of CCR9 led to STAT1 activation (Figure 3H), a response associated with the development of a Th1 phenotype (24).

Optimal expression of α_4_β_7_ by CD4^+^ T cells has been found to be regulated by basic leucine zipper transcription factor, ATF-like (BATF), an AP-1 protein family factor (25). Antibody-activation of CCR9 concomitant to TcR and CD28 engagement induced BATF transcription (Figure 3I) and protein expression by CD4^+^ (Figure 3J) T cells, thus highlighting an additional transcriptional mechanism by which CCR9 signals affect T cell differentiation.

### Ectopic Administration of CCL25 Induces the Differentiation of $\alpha_{4}\beta_{7}^{+}$CCR9^+^ Th1 Cells

CCL25 is not produced in the skin (3, 26) and priming in skin-draining LNs prevents the development of $\alpha_{4}\beta_{7}^{+}$ T cells (27) due to inhibition of RALDH activity by PGE_2_ (28). To rule out that CCL25-mediated accumulation of $\alpha_{4}\beta_{7}^{+}$CCR9^+^-expressing naïve T cells in the GALT prior to priming might underlie the expansion of $\alpha_{4}\beta_{7}^{+}$ T cells, the chemokine was delivered in the subcutaneous tissue together with cognate antigen. To reflect physiologic conditions, the dose of CCL25 was estimated based on the concentration of CCL25 measured in the murine non-inflamed ileum *in vivo* (300 pg/ml) (29), and corrected by the concentration of subcutaneously administered drugs measured in the draining LNs (i.e., 1/1,000 of the injected dose) (30).

**Figure 4:**
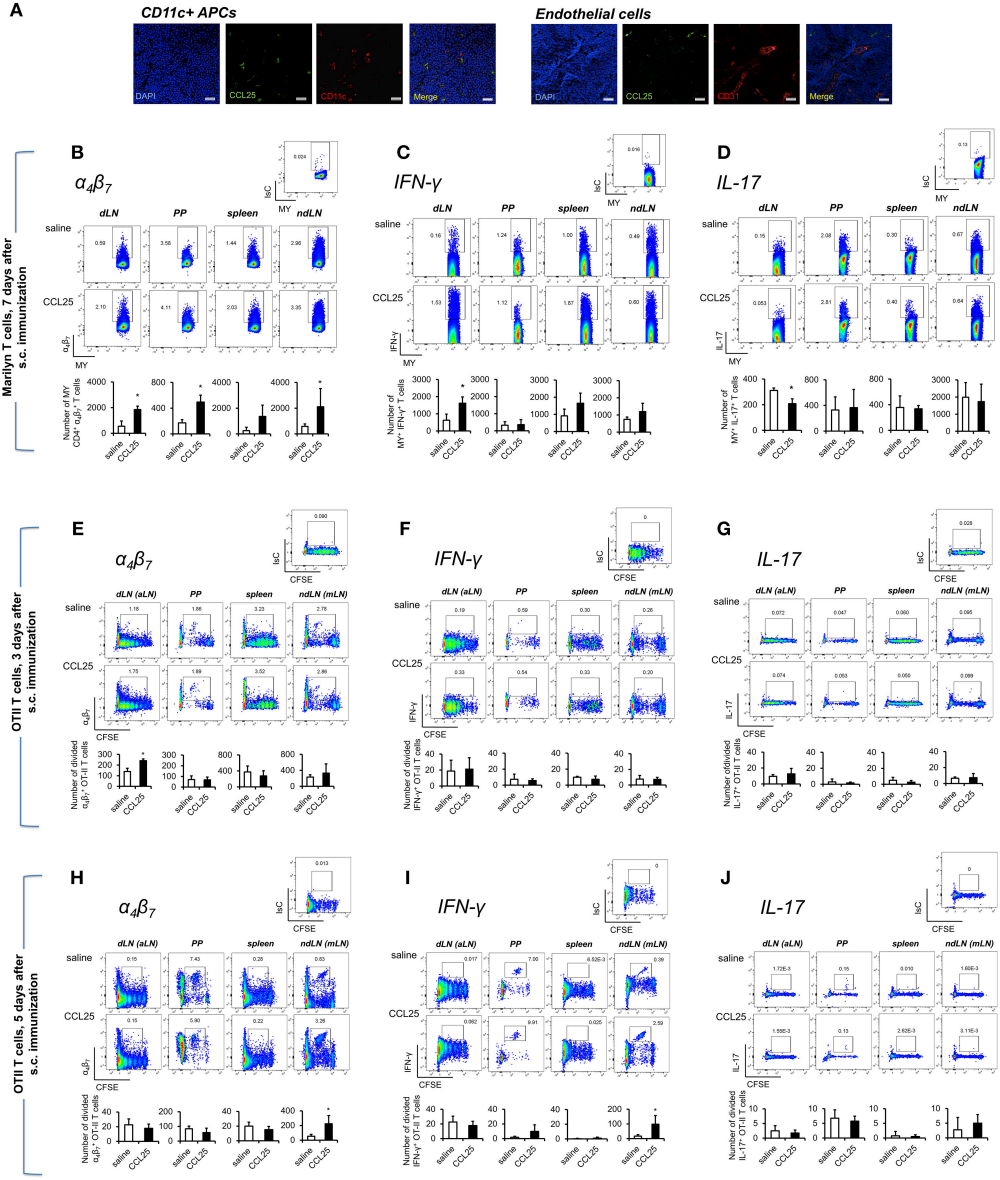
Subcutaneous co-delivery of antigen and CCL25 induces the development of $\alpha_{4}\beta_{7}^{\text{high}}$ Th1 T cells. **(A)** Dylight 488-labeled CCL25 (0.06 mg/kg) was subcutaneously injected into mice. Two hours later, the dLNs were harvested. Tissue sections from dLN were stained with hamster anti-mouse CD11c antibody (clone N418, BioLegend), or rat anti-mouse CD31 Antibody (MEC13.3, BioLegend) overnight at 4°C. Following three washes in PBS, the sections were incubated with the secondary antibody Alexa Fluor® 546 goat anti-hamster IgG or Alexa Fluor® 555 goat anti-rat IgG (Life Technologies) for 30 min at room temperature followed by three washes. Sections were mounted on microscopy slides with 4′,6-diamidino-2-phenylindole (DAPI) mounting medium (Vectashield). Images taken by wide field fluorescence microscopy are shown. Scale bar, 20 μM. **(B–D)** Female Marilyn-*rag2*^−/−^ mice were immunized by subcutaneous administration of 5 × 10^6^ male-derived splenocytes in saline solution or the presence of 0.06 mg/kg CCL25. One week later T cells were separately harvested from axillary (draining LNs, dLN), PP, mesenteric LN (non-draining LN, ndLN), and spleen. Marilyn (MY) T cells were identified by gating on the CD4^+^Vβ6^+^ T cell population. The number of $\alpha_{4}\beta_{7}^{\text{high}}$ **(B)**, IFN-γ- **(C)** or IL-17-expressing **(D)** T cells was measured by flow cytometry. The mean number of T cells from two independent experiments of identical design is shown below each set of dot-plots (±SD, *n* = 3). **(E–J)** OT-II naïve T cells from (10^7^/mouse) were labeled with CFSE (4 μM) and injected intravenously into syngeneic recipients, which were immunized 3 h later by s.c. administration of 0.5 μg OVA-DEC plus 50 μg poly IC adjuvant (InvivoGen) re-suspended in saline solution or in the presence of CCL25 (0.06 mg/kg). T cells were separately harvested from draining LN (axillary, dLN), mesenteric LNs (mLNs), Peyer's Patches (PPs) and spleen 3 **(E–G)** and 5 days **(H–J)** later. Expression of α_4_β_7_ **(E,H)** and IFN-γ **(F,I)** or IL-17 **(G,J)** by CFSE^low^ T cells was assessed by flow cytometry by gating on CD4^+^Vα2^+^ T cells (OTII TCR). Staining with an isotype-matched control antibody in draining lymph nodes of mice injected with adjuvant alone are shown on top of each set of dot plots. The mean values obtained in at least 3 experiments of identical design are shown below each set of representative dot plots (±SD). ^*^p < 0.05.

Dylight 488-labeled CCL25 injected s.c. was detected on CD11c^+^ cells, but not on CD31^+^ vascular structures of draining LNs (Figure 4A). In addition, administration of CCL25 in the absence of antigen did not increase the localization of CCR9^+^ $\alpha_{4}\beta_{7}^{+}$ naïve T cells in the draining LNs compared to injection of saline solution (Supplementary Figures 4A,B).

As shown in Figure 4B, following s.c. immunization of Rag2-deficient Marilyn mice with male-derived splenocytes co-injected with CCL25, a significantly larger population of $\alpha_{4}\beta_{7}^{+}$ CD4^+^ MY T cells became detectable in both draining and non-draining LN, PP and the spleen consistent with the ability of recirculating T cells primed in the skin to rapidly migrate to both local and distal lymphoid tissue (31). CCL25 injection alone did not induce proliferation (not shown). Cytokine analysis revealed an increase of IFN-γ -producing T cells (Figure 4C), which—unexpectedly—was accompanied by a significant decrease of IL-17-producing T cells (Figure 4D). Production of IL-4 was unchanged (Supplementary Figure 4C).

We further explored the differentiation of cytokine-producing CD4^+^ T cells activated in skin-draining LNs in the presence of CCL25 by assessing the expression of α_4_β_7_, CCR9, IFN-γ, and IL-17 by- and the recirculation of- adoptively transferred OTII T cells. $\alpha_{4}\beta_{7}^{+}$ CCR9^+^ OTII T cells differentiated in skin-draining LNs by 3 days after activation (Figures 4E–G and Supplementary Figures 4D,E), and subsequently exited this site and selectively localized to the MLN by day 7 (Figures 4H–J and Supplementary Figures 4D,E), where they had also began to produce IFN-γ (Figure 4I and Supplementary Figure 4F). In this instance, we did not observe increase of gut-homing T cells induced in the presence of CCL25 in all lymphoid sites, possibly reflecting the smaller size of the adoptively transferred TCR-transgenic T cell population compared to that available during immunization of mice with a wholly transgenic TCR repertoire.

**Figure 5:**
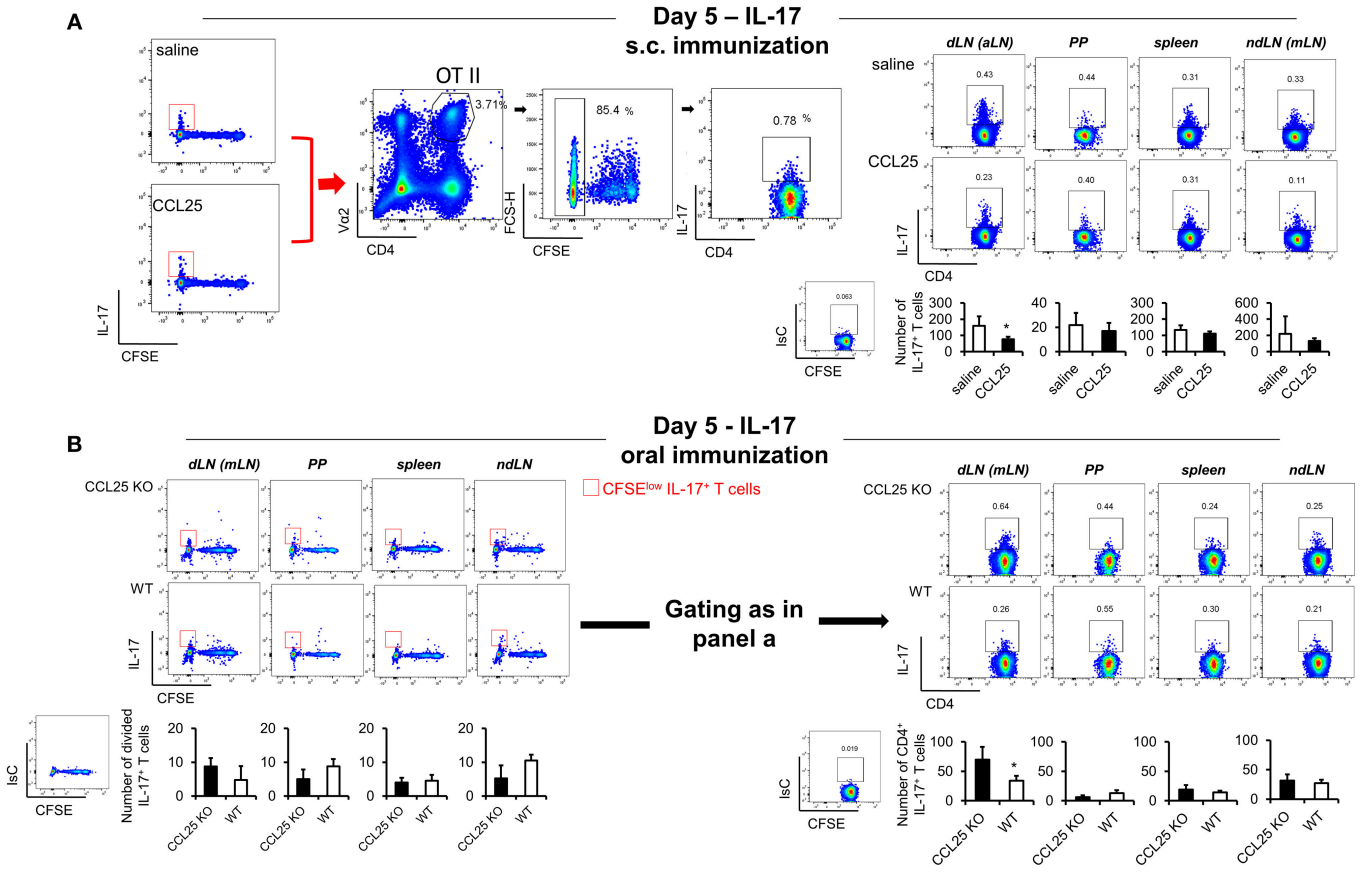
CCL25-induced T_RM_ cells reduce differentiation of IL-17-producing T cells. OT-II naive T cells were labeled with CFSE (4 μM) and injected intravenously into syngeneic recipients (10^7^/mouse), which were immunized 3 h later by s.c. **(A)** or oral **(B)** administration of 0.5 μg OVA-DEC plus 50 μg poly IC adjuvant (InvivoGen) re-suspended in saline solution or in the presence of CCL25 (0.06 mg/kg). T cells were separately harvested from draining LN (axillary, dLN), mesenteric LNs (mLNs), Peyer's Patches (PPs) and spleen 5 days later. Expression of IL-17 by T cells was assessed by flow cytometry by gating on CD4^+^Vα2^+^ (OTII TCR) CFSE^low^ T cells. For clarity, the plots displaying CFSE dilution on divided T cells are shown on the left-hand-side of each panel, in which the red squares indicate the gate used for detection of IL-17^+^ T cells. The mean values obtained in at least three experiments of identical design are shown below each set of representative dot plots (±SD, *n* = 3). ^*^p < 0.05.

In contrast, IL-17-producing T cells did not change in the dividing population containing IFN-γ producing T cells (Figures 4G,J). However, we noticed that the number of IL-17-producing OTII T cells (Figure 5A) was significantly decreased in the CFSE^low^ T cells 5 days after immunization, suggesting the possibility that CCL25 affects the differentiation of IL-17^+^ OT-II T cells from naïve precursors distinct from those generating $\alpha_{4}\beta_{7}^{+}$ IFN-γ-producing T cells. Furthermore, IL-17^+^ OT-II T cells were mainly detected in skin dLN and did not localize to the GALT. Analysis of OT-II T cells retrieved in the dLN following oral immunization of WT and CCL25-deficient mice confirmed that IL-17-producing T cells preferentially expand in the gut in *ccl25*^−/−^ animals (Figure 5B).

### CCL25-Induced Memory T Cells Localize to the Gut Wall and Peyer's Patches and do Not Recirculate Upon Remote Antigen Challenge

Differentiation $\alpha_{4}\beta_{7}^{+}$ T cells induced by CCR9 signals *in vivo* consistently occurred in a small population of activated T cells (Figure 2) suggesting that only a fraction of TCR-transgenic CCR9^+^ T cells undergoes such differentiation pathway. The generation of these T cells by s.c. immunization in the presence of CCL25 provided the opportunity to further characterize this population without the need to discriminate them from other $\alpha_{4}\beta_{7}^{+}$ CCR9^+^ T lymphocytes already present in the GALT microenvironment.

**Figure 6:**
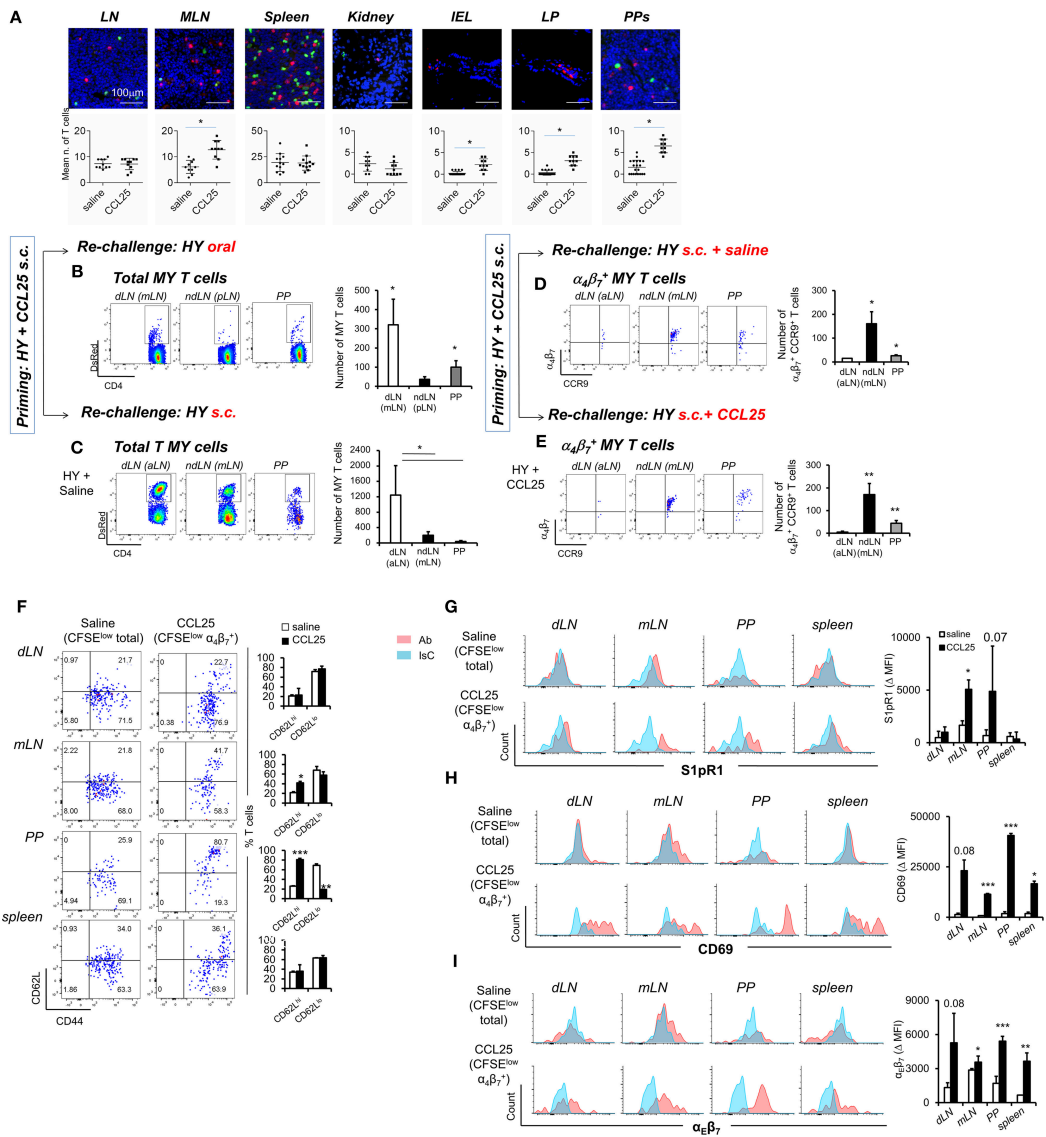
CCL25-induced $\alpha_{4}\beta_{7}^{+}$ CD4^+^ T cells localize to the GI tract and do not recirculate. T cells isolated from dLN of Marilyn-*rag2*^−/−^ mice immunized s.c. with male splenocytes either in the presence or absence (saline) of the chemokine CCL25 (0.06 mg/kg) were labeled with the red-fluorescence cell-linker PKH26 (2 μM) and injected i.v. (10^7^/mouse) into syngeneic C57BL/6 male recipients. Localization of labeled T cells in the indicated tissues was analyzed 24 h later by wide field fluorescence microscopy. Representative images and the mean number of labeled cells detected in at least three samples from each recipient in three independent experiments (*n* = 3) are shown in **(A)**. Scale bar: 300 μm. CD44^hi^ DsRED-expressing Marilyn T cells were isolated by cell sorting from the dLN (axillary) and spleen of mice immunized s.c. 7 days earlier in the presence of CCL25. **(B,C)** T cells were subsequently transferred into naïve female C57BL/6 recipients, which received an antigen challenge in saline solution orally **(B)** or s.c. **(C)** a week later. The presence of DsRED Marilyn (MY) T cells in the dLNs, ndLN and PPs was assessed 4 days after antigenic challenge. MY T cells were further identified by the expression of Vβ6. The mean number of T cells from at least three recipients (±SD) is shown on the right-hand-side of representative dot-plots (^*^p < 0.05). **(D,E)** Recipients of T cells from mice primed s.c. in the presence of CCL25 were also antigen-re challenged s.c. in the absence or presence of CCL25. To discriminate the $\alpha_{4}\beta_{7}^{+}$ and CCR9^+^ cells within the MY CD4^+^ T cell population gating was set on DsRED and CD4. DsRED Marilyn (MY) T cells in the dLNs, ndLN, and PPs were analyzed 4 days after antigenic challenge. The mean number of T cells from at least three recipients (±SD) is shown on the right-hand-side of representative dot-plots (^*^p < 0.05, ^**^p < 0.01). **(F–I)** CD4^+^ T cells from Marilyn-*rag2*^−/−^ mice (10^7^/mouse) were labeled with CFSE (4 μM) and injected intravenously into female syngeneic recipients. Recipient mice were immunized 24 h later by s.c. administration of 5 × 10^6^ male-derived splenocytes re-suspended in saline solution or in the presence of CCL25 (0.06 mg/kg). One week later, T cells were separately harvested from draining LN (axillary, dLN), mesenteric LNs (mLNs) and Peyer's Patches (PPs) and spleen. Expression of the indicated molecules was assessed by flow cytometry. Comparison are made on Marilyn T cells identified by gating on the total divided CD4^+^Vβ6^+^CFSE^low^ T cell population from mice immunized in the presence of saline, and on the CD4^+^Vβ6^+^${\text{CFSE}}^{\text{low}}\alpha_{4}\beta_{7}^{\text{high}}$ T cell population from mice immunized in the presence of CCL25. CD4^+^Vβ6^+^${\text{CFSE}}^{\text{low}}\alpha_{4}\beta_{7}^{\text{high}}$ T cells were retrieved in negligible numbers from mice immunized in the presence of saline, making it unfeasible to analyze this population. In panel **(F)**, representative dot plots are sided by graphs representing the mean % of T cells detected in at least three animals, In panels **(G–I)**, representative histograms of CCL25-induced or control T cells are shown. The blue histograms depict staining with an isotype-matched control antibody (IsC). In each panel, the column graphs display the mean delta MFI (experimental MFI—IsC MFI) detected in T cells from three animals (±SD, ^*^p < 0.05, ^**^p < 0.01, ^***^p < 0.001) (*N* = 2).

First, we sought to confirm that T cells primed in the presence of CCL25 in the subcutaneous dLN develop topographic memory for the GI tract and associated lymphoid tissue and to define their distribution within this organ. CD44^high^ T cells were isolated from dLNs of Marilyn-*rag2*^−/−^ mice immunized subcutaneously (s.c.) either in the presence or absence of CCL25, then labeled and adoptively transferred into syngeneic C57BL/6 male recipients. T cell localization in a number of sites was analyzed 24 h later by wide field fluorescence microscopy. As shown in Figures 6A–C, adoptively transferred T cells activated in the presence of CCL25 preferentially localized to MLNs and PP compared to those primed in the presence of saline solution. Strikingly, only T cells activated in the presence of CCL25 gained access to the gut wall (Figure 6A and Supplementary Figure 5), where they localized in the LP as well as in the epithelial layer.

Second, we assessed the ability of these T cells to recirculate by delivering a remote antigen challenge. Primed CD44^high^ fluorescent TCR-transgenic T cells were isolated from the spleen and dLN of DsRed-expressing Marilyn-Rag2^−/−^ mice immunized s.c. with male splenocytes and CCL25 a week earlier. Following isolation from LN and spleen, T cells were adoptively transferred into naïve female C57BL/6 recipients. A week later, mice were challenged with antigen (Dby peptide) either orally (Figure 6B) or s.c. (Figure 6C) without co-injection of CCL25. The presence of fluorescent MY T cells in the draining and non-draining LNs and PPs was assessed for up to 4 days later. Following oral re-challenge the majority of DsRed MY T cells converged to the MLN and PP (Figure 6B), irrespectively of expression of α_4_β_7_ and/or CCR9. Similarly, antigenic challenge s.c. led to T cell localization to the draining axillary LNs (Figure 6C), suggesting that the majority of T cells primed in the presence of CCL25 recirculate and migrate to remote sites of antigen challenge. However, the $\alpha_{4}\beta_{7}^{+}$CCR9^+^ T cell subset induced by s.c. immunization with CCL25 remained localized to the mLN and PP upon remote (s.c.) antigen administration (Figure 6D). Even co-delivery of the chemokine CCL25 during antigen re-challenge s.c. did not elicit migration of these T cells to the skin-draining LNs (Figure 6E), suggesting that CCL25-induced $\alpha_{4}\beta_{7}^{+}$CCR9^+^ T cells do not recirculate once localized in the gut wall and associated lymphoid tissue.

We therefore analyzed the expression of receptors associated with lymphoid and non-lymphoid tissue retention signals by $\alpha_{4}\beta_{7}^{+}$CCR9^+^ MY T cells, which had divided (i.e., CFSE-low) upon s.c. immunization in the presence of CCL25 a week earlier. As a control, the phenotype of MY T cells divided following immunization in saline solution was analyzed. As shown in Figure 6F, a larger proportion of $\alpha_{4}\beta_{7}^{+}$CCR9^+^ MY T cells displayed CD62L compared to $\alpha_{4}\beta_{7}^{-}$CCR9^−^ divided MY T cells. Both T cell populations expressed the S1PR1 (Figure 6G), a key receptors necessary for T cell egress from lymph nodes (32). However, only $\alpha_{4}\beta_{7}^{+}$CCR9^+^ T cells expressed the S1PR1-blocking molecule CD69 (Figure 6H) (33), which is required for the retention of T cells in the PPs (34), and α_E_β_7_ integrin (Figure 6I), which mediates adhesion of T cells to the epithelium (35) and is characteristically expressed by IELs (1). Thus, CCL25-induced $\alpha_{4}\beta_{7}^{+}$CCR9^+^ T cells display phenotypic features similar to those of T_RM_ cells.

### CCL25-Induced Th1 Cells Regulate the Tissue Immune Environment

We finally tested the hypothesis that the ability of CCL25-induced $\alpha_{4}\beta_{7}^{+}$CCR9^+^ mucosal T cells to influence cytokine production might enable them to define the tissue microenvironment. As strong Th1 responses are bound to occur during most bacterial GI infections rendering it difficult to discriminate the role of this small population from overall immune activation we first assessed the impact of CCL25-deficiency in a model of *N. brasiliensis* infection in mice, in which resistance is dependent on CD4^+^ T cells of the Th2 type (31).

**Figure 7:**
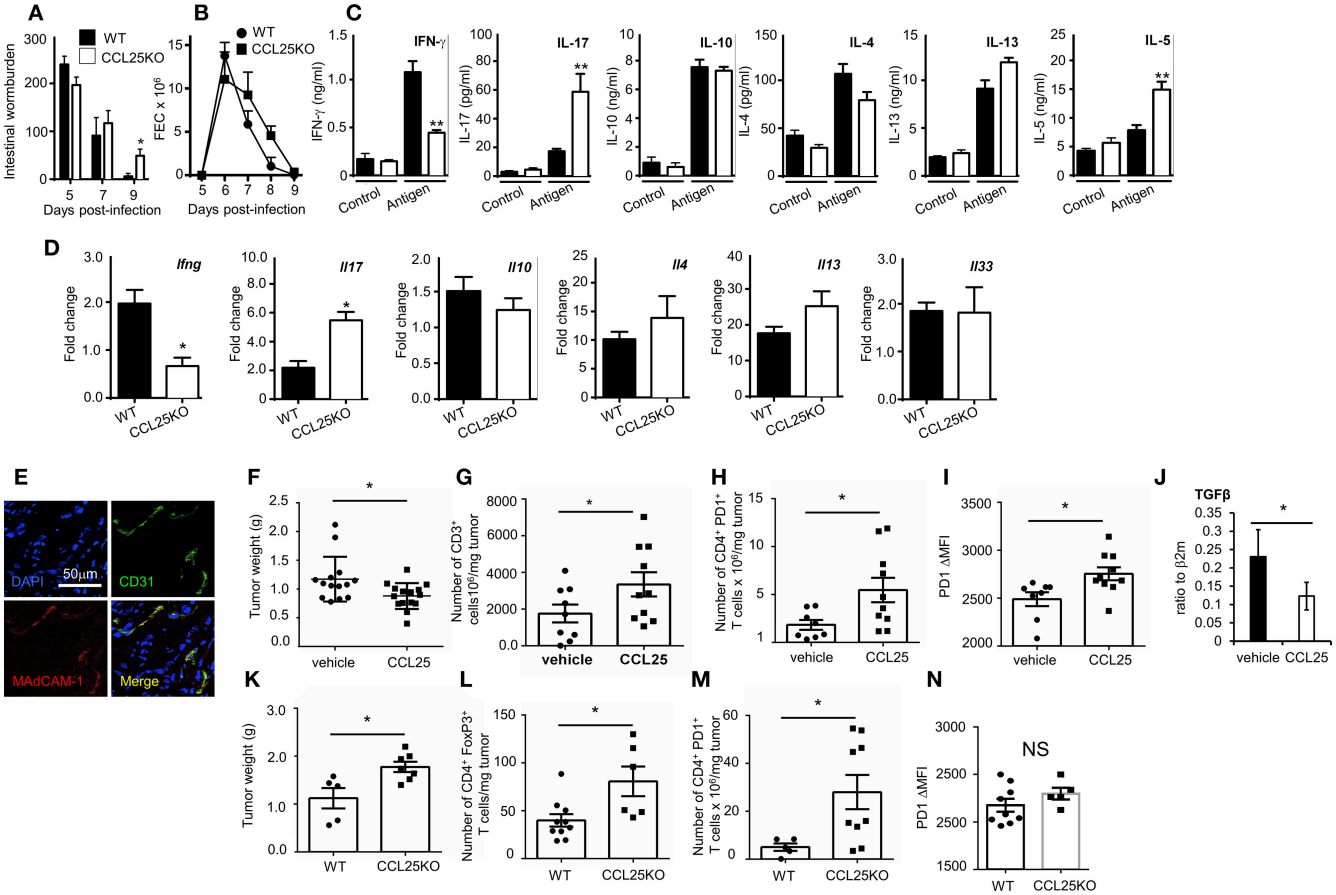
CCL-induced T_RM_ cells affect T cell responses by modifying the immune environment. Intestinal wormburden and fecal expulsion of helminth by WT mice and *ccl25*^−/−^ infected with *N. brasiliensis* are shown in panels **(A)** and **(B)**, respectively. In panel **(C)**, production of the indicated cytokine from *in vitro* worm antigen-stimulated mesenteric lymph node cultures. Expression of IFN-γ, IL-17, IL-10, IL-4, IL-13, and IL-33 mRNA in intestinal tissue from infected mice is shown in panel **(D)**. *n* = 5 in each group. Data are representative of two independent experiments (mean ± SEM, ^*^p < 0.05). Expression of MAdCAM-1 molecules by vascular endothelium in orthotopic PDAC is shown in panel **(E)**. Mice were immunized with tumor cell lysates and syngeneic splenocyte with and without CCL25 a week before receiving intrapancreatic tumor cell implants. Panel **F**, weight of tumors excised 28 days after implantation. Tumor immune cell infiltrates were subsequently analyzed for the presence of CD3^+^ T cells **(G)** CD4^+^PD1^+^ T cells **(H)** and intensity of PF1 expression on these cells **(I)**. The presence of Tgfβ mRNA was assessed by RT-PCR **(J)**. In parallel experiment WT and CCL25-deficient mice were immunized with tumor cells and syngeneic splenocyte lysates a week before receiving intrapancreatic tumor cell implants. Panel **(K)**, weight of tumors excised 28 days after implantation. Tumor immune cell infiltrates were subsequently analyzed for the presence of CD4^+^FoxP3^+^ cells **(L)**, CD4^+^PD1^+^ cells **(M)** and intensity of PF1 expression on these cells **(N)** *n* > 5 *N* = 4 ^*^p < 0.05; ^**^p < 0.01.

WT and CCL25-deficient mice were infected with embryonated *N. brasiliensis* eggs, and worm burdens, fecal egg count (FEC) and cytokine responses *ex vivo* were analyzed at specific time points post infection. As it is shown in Figures 7A–C, lack of CCL25 resulted in a relatively small but significant delay in worm clearance, which was associated with reduced IFN-γ and increased IL-17 and IL-5 production in the local lymph nodes, whilst and IL-4 and IL-13 secretion was unaffected. Intestinal mRNA expression analysis confirmed that lack of CCL25 resulted in significantly decreased IFN-γ and increased IL-17 mRNA expression in the gut (Figure 7D). No change in IL-33 mRNA expression was observed, suggesting that CCL25 deficiency unlikely affects epithelial cells.

Having established that the CCL25/CCR9 axis influences physiological immunity in the intestine by cytokines, we investigated whether induction of the Th1 $\alpha_{4}\beta_{7}^{+}$ population could also modulate tissue immune cell infiltrates, as it has been shown for T_RM_ (9, 36). We chose to assess the effect of co-delivering immunogen and CCL25 on anti-tumor immune responses in a model of orthotopic PDAC model. In the PDAC microenvironment, the prevalent immune cells tending to promote tumor progression via immunosuppression include T regulatory cells (T_reg_), Myeloid Derived Suppressor Cells and Type 2 Tumor Associated Macrophages, while dampening immune effector cells (mainly CD8^+^ and CD4^+^ T cells, Dendritic Cells (DCs) and Natural Killer Cells) (37). We confirmed previous reports in humans that Mucosal Addressin Cell Adhesion Molecule (MAdCAM)-1, which enables access by $\alpha_{4}\beta_{7}^{+}$ T cells is expressed at high levels by the vascular endothelium of PDAC (Figure 7E) but not of adjacent tissue (38). Mice were immunized intraperitoneally with tumor cell lysates in the presence or absence of CCL25 a week before receiving intrapancreatic PDAC cell injection, having previously confirmed that also this immunization route in the presence of CCL25 induces the differentiation of $\alpha_{4}\beta_{7}^{+}$ Marilyn T cells (Supplementary Figure 6). Mice were sacrificed 28 days after tumor implantation, and the tumor was weighted and processed for analysis. As shown in Figure 7F, tumor growth was significantly reduced in mice previously immunized in the presence of CCL25. A detailed analysis of immune cell infiltrates showed a significant increase of CD3-expressing cells (Figure 7G) without increase of CD4^+^ or CD8^+^ populations (Supplementary Figures 7A,B). In addition, a significantly larger number of T cells expressed higher levels of PD-1 (Figures 7H,I), without increase of CD4+ FoxP3+ T cells (Supplementary Figure 7C), indicating immune activation. These tumors contained higher numbers of inflammatory monocytes, macrophages and dendritic cells, while B-cell and granulocyte numbers remained unchanged (Supplementary Figures 7D–H). We did not detect changes in the production (Supplementary Figures 7I,J) or transcription of IL-17 and IFNγ (Supplementary Figure 7K), but detected a significant decrease in TGFβ mRNA (Figure 7J).

As the PDAC cell line was found to spontaneously produce CCL25 (Supplementary Figure 8A), and delivery of chemokine to tumor-bearing, non-immunized mice was not effective due to the extremely fast tumor growth, we further sought to rule out the role of chemotactic activity by the tumor itself and to assess its expansion in recipient unable to produce CCL25. To this aim, tumor growth and immune-environment characteristics were compared in WT and *ccl25*^−/−^ immunized recipients (without CCL25). As shown in Figure 7K tumor growth was increased in CCL25-deficient recipients, and a detailed analysis of immune cell infiltrates revealed a significantly increased number of FoxP3 CD4^+^ T cells (Figure 7L), which was accompanied by a significantly larger number of PDI-expressing cells (Figure 7M) but without upregulation of this molecule on a per cell basis (Figure 7N). Analysis of cell infiltrates revealed an overall decrease in inflammatory cells and cytokines, with no changes in *Tgfb* transcripts (Supplementary Figures 8B–N). Collectively, these data show that CCL25 physiologically promotes anti-tumor responses in the GI system by modifying the immune microenvironment rather than by means of its chemotactic activity.

## Discussion

This study describes a Th1 population, which develops as a result of CCR9 signals delivered during priming, maintains expression of α_4_β_7_ integrin, display phenotypic features similar to those of T_RM_ and localizes in the gut wall and GALT.

The concept that mucosal immune surveillance is solely carried out by circulating $\alpha_{4}\beta_{7}^{\text{high}}$ memory T cells, has been challenged by the observation that α_4_β_7_ integrin is lost by the majority of recirculating memory T cells—including those activated in the GALT—despite the maintenance of protective immunity (39). However, while expression of α_4_β_7_ integrin is not required for efficient T cell entry in the intestine and pathogen clearance during primary infections, α_4_β_7_ integrin is crucial for protection against subsequent infection. The development of mucosal α_4_β_7_ T_RM_ cells has been recently confirmed to be necessary for optimal pathogen clearance in a model of genital Chlamydia trachomatis infection (40). Together with this evidence, our own findings indicate that following priming α_4_β_7_ persists only in a small, discrete population of Th1 cells, suggesting that α_4_β_7_ expression is essential for the localization and retention of mucosal T cells. Although parabiosis experiments will be required to formally define the CCL25-induced Th1 cells that we describe here as T_RM_ cells, their phenotypical feature, mucosal and epithelial localization, lack of recirculation upon remote antigen challenge and long-term effect on the immune microenvironment in tumors suggest that these cells are functionally more similar to T_RM_ cells than those activated in the absence of CCL25.

Similarly to IELs, CCL25-induced mucosal Th1 cells express α_E_β_7_, which might facilitate their adhesion to the gut epithelium (35), as well as other typical markers of T_RM_ cells. Importantly, this subset specializes in the production of IFN-γ. T_RM_ cells have been shown to exert their protective role by producing cytokines including IFN-γ (41, 42), which serve to enhance expression of adhesion molecules by the vascular endothelium and subsequently promote recruitment and activation of other immune cells (43). In line with these reports, we show that a key function of CCL25-induced Th1 T_RM_ cells is to modulate the immune microenvironment and ultimately the immune response in an indirect manner, by regulating the recruitment and activation of other immune cells (as we have observed in the PDAC model), or their function (such as in the parasite infestation model). This concept is best illustrated by the recent report that antigenic re-challenge of T_RM_ cells in the skin can induce a non-antigen-specific state of alert, with increased tissue-wide expression of a substantial number of innate immune response genes, including interferon-induced transmembrane protein-3 (12). These findings also provide an explanation for the paradox that, despite displaying only minor quantitative defects in gut IELs, genetic ablation of the CCR9/CCL25 axis in mice leads to exacerbation of intestinal T cell responses, accompanied by increased IL-17 production (15, 44, 45). Consistently with these observations, we show that the development of CCL25-induced mucosal Th1 cells correlates with a decrease of IL-17-producing T cells from distinct naïve T cell precursors both *in vitro* and *in vivo*. The molecular mechanism of this effect is at present unclear, but it is likely to be related to the impact of CCL-25 induced mucosal Th1 cells on the immune microenvironment, as suggested from our studies in the helminth infestation and PDAC models.

The transcriptional regulation of T_RM_ cell has recently received much attention (9), especially in view of the identification of phenotypically and functionally distinct Th17- and Th1-like T_RM_ cells in the skin (46), however little is known about the stimuli that regulate their differentiation. Our observations indicate that signals initiated by the CCL25/CCR9 axis induce mucosal Th1 cells. Specifically, CCR9 signals favor the induction of a transcriptional program which includes T-bet and BAFT expression. With respect to T-bet expression, CCR9 triggering might aid optimal positioning of naïve CCR9^+^ T cells during priming as it has been shown for CXCR3 (47) and/or stabilize cognate interactions with antigen-presenting DCs, thus strengthening TCR signals and leading to Th1 differentiation (48). Alternatively, or in addition, CCR9 signals might directly influence T-bet expression by converging signals on the Jak-STAT pathway (49). CCL25 might also enhance Th1 response in an indirect manner, by enhancing their survival via Akt activation. Further investigations are required to fully define this mechanism.

Our observations further indicate that the CCR9-induced transcriptional program leading to the differentiation of mucosal Th1 cells is initiated during activation in the lymph nodes and prior to their localization to the gut wall and GALT. The failure by CCL25 to induce α_4_β_7_ TRM T cells in CCR9-deficient T cells (unlike RA) and the detection of this small cohort of T cells in a discrete population of divided T cells could be explained by the existence of a pre-committed CD4^+^ CCR9^+^ $\alpha_{4}\beta_{7}^{+}$ precursor naïve T cell subset. Alternatively, or in addition, specialized differentiation—in this case α_4_β_7_ expression and IFN-γ production—might occur in a cohort of activated T cells upon very stringent conditions, such as the strength of TCR signals (50) in conjunction with CCR9 signals. In this instance, specialized differentiation is reflected by a specific number of cell divisions undergone by the differentiated T cells (50), as we have consistently observed in TCR-transgenic populations activated *in vivo* in the presence of CCL25, independently of the immunization route. In support of this hypothesis, a larger cohort of T cells activated *in vitro* by CD3 stimulation—likely to engage the majority of T cells—upregulated α_4_β_7_ expression in the presence of CCL25.

In conclusion, our investigation provides proof-of-concept that induction of specific subsets of mucosal T cells can be achieved by remote immunization, thus paving the way for the therapeutic manipulation of mucosal immunity.

## Ethics Statement

This study was carried out in accordance with the recommendations of the Home Office of the UK. The protocol was approved by the Queen Mary University of London committee.

## Author Contributions

HF and HH designed and performed experiments, analyzed and interpreted data, and wrote the manuscript. MJ, AP, GW, DC, and SS designed and performed experiments and contributed to data interpretation and discussion. IS and MaC provided reagents. GC performed experiments. MeC contributed to discussions. FM-B designed experiments, interpreted data, and wrote the manuscript.

### Conflict of Interest Statement

The authors declare that the research was conducted in the absence of any commercial or financial relationships that could be construed as a potential conflict of interest.
