# Supplementary material for: A Subset of CCL25-Induced Gut-Homing T Cells Affects Intestinal Immunity to Infection and Cancer
Source: Front Immunol. 2019 Feb 25;10:271. doi: 10.3389/fimmu.2019.00271 (PMC6400137; doi:10.3389/fimmu.2019.00271)
Supplement: Supplementary file 1 [file Data_Sheet_1.docx]

**SUPPLEMENTAL FIGURES.**

**Supplemental Figure 1.**

**CCL25 induces the acquisition of gut-homing receptors during in vitro T cell activation.**

(**A**) Expression of CCR9 by purified CD4^+^ and CD8^+^ naïve T cells.

(**B**) Naïve T cells were purified by immunomagnetic selection. Routinely, more than 97% of the purified cells were CD44^low^ CD62^high^.

(**C-D**) Purified naïve T cells from WT and *ccr9^-/-^* were activated with an anti-CD3 (2C11, 1µg/ml) monoclonal antibody and syngeneic DC differentiated in the presence of the RALDH inhibitor citral (0.1µM) (medium). In some cultures, CCL25 was added in soluble form (300ng/ml) or via DC pre-incubated with the chemokine (CCL25-DC) for 40 minutes followed by washing in PBS. In addition, T cells were activated in the bottom chamber of a transwell in medium containing CCL25 in the presence of DCs seeded on the upper chamber (separated). Non stimulated naïve T cells (NS) or T cells exposed to all-trans retinoic acid (RA) were analyzed as a control. After 5 days expression of the integrin α_4_β_7_ by CD3^+^ T cells was assessed by flow cytometry. Non-specific staining by an Isotype-matched Control Ab (IsC) was assessed on a mixture of WT and *ccr9^-/-^* activated T cells. Panel **D** shows cumulative data in a representative of 3 experiment of identical design each performed in triplicate (±SEM).

**(E)** BM-DCs were either left untreated or cultured with 0.1µM Citral. The RALDH inhibitor diethylaminobenzaldehyde (DEAB) was used as negative control. RALDH activity was measured with the ALDEFLUOR reagent followed by detection by flow cytometry. In panel a, a representative histogram is shown. The mean RALDH activity detected in 3 experiments of identical design is shown in Panel B. ****p<0.0001

**Supplemental Figure 2.**

**Physiologic localization of CCL25 *in vivo* and role of CCR9 signaling.**

Tissue sections from PP, *lamina propria* (LP), mesenteric LNs (mLN) and inguinal (i) LN of naïve mice either untreated (Steady State) or 48 hours after oral administration of 50μg of CpG ODN (Adjuvant) were stained with rat anti-mouse CCL25 Ab (Clone 89827, R&D), hamster anti-mouse CD11c antibody (clone N418, BioLegend) (panels **A, C**), goat anti- mouse CD103 (R&D, AF1990-SP) (**C**) or polyclonal rabbit anti-mouse CD31 antibody (Abcam) (panels **B, D**) overnight at 4°C. Following three washes in PBS, the sections were incubated with the secondary antibody Alexa Fluor® 555 goat anti-rat IgG or Alexa Fluor® 488 goat anti-Hamster IgG, or Alexa Fluor® 488 goat anti-Rabbit IgG (Life Technologies) for 30 min at room temperature followed by 3 washes. Sections were mounted on microscopy slides with 4',6-diamidino-2-phenylindole (DAPI) mounting medium (Vectashield). Images were taken by wide field fluorescence microscopy are shown. Scale bar, 10 µm.

(E) TCR-transgenic, naïve MY T cells (10^7^/mouse) were labelled with CFSE (4µM) and injected intravenously into WT and *ccl25^-/-^* syngeneic female recipients. 24 hours later, recipient mice received cognate peptide (100µg Dby peptide) plus ODN adjuvant (50µg) orally. A week after immunization, T cells were separately harvested from mesenteric LN (draining LN, dLN), Peyer’s Patches (PP), inguinal and axillary (non-draining LNs, ndLN) and the spleen.

Co-expression of α_4_β_7_ and CCR9 by divided T cells was assessed by flow cytometry. The mean number of divided α_4_β_7_^+^ and CCR9^+^ T cells from 3 independent experiments of identical design is shown below a set of representative dot plots (± SD). *p<0.05

(F) WT and CCR9-KO naïve T cells (10^7^/mouse) were labelled with CFSE (4µM) and injected intravenously into syngeneic female recipients. On day 1 and day 3, recipient mice received a mixture of allogeneic BALB/C and Cba/Ca splenocytes (60x10^6^/mouse) plus ODN adjuvant (100µg) orally. One week later, T cells were separately harvested from mesenteric LN (draining LN, dLN), Peyer’s Patches (PP), inguinal and axillary (non-draining LNs, ndLN) and the spleen. Expression of α_4_β_7_ by divided WT and *ccr9^-/-^* T cells was assessed by flow cytometry (by gating on the CD3^+^ population). The mean percentage of divided α_4_β_7_ T cells from 2 independent experiments of identical design is shown below each set of representative dot plots in panel (± SD, *p<0.05). n=3

**Supplemental Figure 3.**

**CCR9 signals induce α_4_β_7_^+^ Th1-like cells.**

OT-II naïve T cells (MY 10^7^/mouse) were labelled with CFSE (4µM) and injected intravenously into WT and *ccl25^-/-^* syngeneic recipients. 24 hours later, recipient mice received cognate peptide (0.5μg OVA-DEC peptide) plus ODN adjuvant (50µg) orally. Five days later, T cells were separately harvested from mesenteric LN (draining LN, dLN), inguinal and axillary (non draining LNs, ndLN) and the spleen. Production of IL-4 (**A**) and IL-17 (**B**) by divided T cells was assessed by intracellular staining and flow cytometry. The mean percentage of α_4_β_7_^+^ T cells from 2 independent experiments is shown below representative dot plots (± SD, n=3).

(C) Female Marilyn mice were immunized by i.p. administration of 5x10^6^ male-derived splenocytes either resuspended in saline solution in the presence of an activating anti-CCR9 antibody (1mg/kg) or an Isotype-matched antibody control (IsC). Seven days later T cells were separately harvested from dLN (mLn), PPs, spleen and ndLN. Expression of the gut-homing receptors α_4_β_7_ was assessed by flow cytometry. Panel **D** summarizes the mean data from 2 experiments of identical design (± SD, *p<0.05).

**Supplemental Figure 4.**

**Administration of CCL25 does not induce preferential accumulation of α_4_β_7_^+^ or CCR9^+^ naïve T cells in secondary lymphoid tissue.**

Female Marilyn mice received 0.06 mg/kg CCL25 or saline solution by s.c. administration. After 16 hours T cells were separately harvested from dLN, ndLN and spleen. The presence of CCR9^+^ (**A**) or α_4_β_7_^+^ (**B**) T cells was quantified by flow cytometry. The right-hand panels show the mean data from 3 recipients in a representative experiment (± SD, N=2).

(**C**) Marilyn CD4^+^ T cells from OT-II (10^7^/mouse) were labeled with CFSE (4µM) and injected intravenously into syngeneic recipients, which were immunized 3 hours later by s.c. administration of male-derived splenocytes re-suspended in saline solution or in the presence of CCL25 (0.06 mg/kg). T cells were separately harvested from draining LN (axillary, dLN), mesenteric LNs (ndLNs), Peyer’s Patches (PPs) and spleen 5 days later. T cells were subsequently stimulated *in vitro* with male DCs. The percentage of IL-4-producing T cells was assessed by intracellular cytokine staining and flow cytometry. Mean data from 3 recipients in a representative experiment are shown below each set of dot-plots.

(**D-F**) OT-II CD4^+^ T cells from (10^7^/mouse) were labeled with CFSE (4µM) and injected intravenously into syngeneic recipients, which were immunized 3 hours later by s.c. administration of 0.5ug OVA-DEC plus 50 µg poly IC adjuvant (InvivoGen) re-suspended in saline solution or in the presence of CCL25 (0.06 mg/kg). T cells were separately harvested from draining LN (axillary, dLN), mesenteric non-draining LNs (ndLNs), Peyer’s Patches (PPs) and spleen 3 and 5 days later. T cells were subsequently stimulated *in vitro* with DCs pulsed with OVA (10μg/ml). The presence of CCR9^+^ T cells 3 days (**A**) or 5 days (**B**) after immunization was quantified by flow cytometry. (**F**) The percentage of IFN-γ-producing α_4_β_7_^+^ T cells was assessed by intracellular cytokine staining and flow cytometry. OTII T cells were identified by gating on the CD4^+^Vα2^+^ T cell population. Cumulative data from 3 recipients in a representative experiment are shown below each set of dot-plots (± SD, *p<0.05, **p<0.01; N=3).

**Supplemental Figure 5.**

**α_4_β_7_^+^ Th1-like cells localize in the intra-epithelial and *lamina propria* areas.** T cells isolated from dLN of Marilyn-Rag2^-/-^ mice immunized s.c. either in the presence or absence (saline) of the chemokine CCL25 (0.06 mg/kg) were labelled with the red-fluorescence cell-linker PKH26 (2µM) and injected i.v. (10^7^/mouse) into syngeneic C57BL/6 male recipients. Localization of labelled T cells in the indicated tissues was analyzed 24 hours later by wide field fluorescence microscopy. Representative images at 10x magnification are shown, with squares highlighting T cells positioned within the epithelial layer (A) or the villi LP (B). Scale bar: 300μm

**Supplemental Figure 6.**

**Intraperitoneal Immunization in the presence of CCL25 induces α_4_β_7_^+^ Th1-like cells.** Female Marilyn-Rag2^-/-^ mice were immunized by intraperitoneal administration of 5x10^6^ male-derived splenocytes in saline solution or the presence of 0.06 mg/kg CCL25. One week later T cells were separately harvested from axillary (draining LNs, dLN), PP, mesenteric LN (non-draining LN, ndLN), and spleen. Marilyn (MY) T cells were identified by gating on the CD4^+^Vβ6^+^ T cell population. The number of α_4_β_7_- (**A**), and CCR9-expressing (**B**) T cells was measured by flow cytometry. The mean number of divided α_4_β_7_ T cells from 2 independent experiments of identical design is shown.

**Supplemental Figure 7**

**Characterization of PDAC immune environment after vaccination with or without CCL25.** Mice were immunized with tumor cell and syngeneic splenocyte lysates with and without CCL25 a week before receiving intrapancreatic tumor cell implants. Tumor immune cell infiltrates were analyzed 28 days after implantation for the presence of CD4^+^ (A), CCD8^+^ (B), FoxP3^+^ (C), CD19^+^ (D), Ly6G^+^ (E), Ly6Cbright (F), F4/80^+^ (G), CD11c^+^ (H), IL-17^+^ (I) cells, and percentage of CD4^+^IFNγ^+^ cells (J). The presence of IFNγ and IL-17 mRNA was also assessed by RT-PCR (K). n>5, N=3

**Supplemental Figure 8**

**Characterization of PDAC immune environment in immunized CCL25-competent or -deficient hosts.** (**A**) tumor cells were stained with rat anti-mouse CCL25 Ab (Clone 89827, R&D) overnight at 4°C. Following three washes in PBS, the cells were incubated with the secondary antibody Alexa Fluor® 555 goat anti-rat IgG (Life Technologies) for 30 min at room temperature followed by 3 washes. Cells were mounted on microscopy slides with 4',6-diamidino-2-phenylindole (DAPI) mounting medium (Vectashield) for imaging.

(**B–N**) WT and CCL25-deficient mice were immunized with tumor cell and syngeneic splenocyte lysates a week before receiving intrapancreatic tumor cell implants. Tumor immune cell infiltrates were analyzed 28 days after implantation for the presence implantation for the presence of CD3^+^ (B), CCD4^+^ (C), CD8^+^ (D), CD19^+^ (E), Ly6G^+^ (F), Ly6Cbright (G), F4/80^+^ (H), CD11c^+^ (I), IL-17^+^ (J) cells, and percentage of CD4^+^IFNγ^+^ cells (K). The presence of IFNγ, IL-17 and TGFβ mRNA was also assessed by RT-PCR (L-N). n>5 N=4
